# Human growth hormone, a marker for HIV infection among adult Igbo Nigerians: relationship between human growth hormone and CD4+ count with viral load

**DOI:** 10.4314/ahs.v23i2.10

**Published:** 2023-06

**Authors:** Collins U Obi, Peter U Achukwu, Chukwugozie N Okwuosa, Okikioluwa S Aladeyelu, Ijeoma N Agbiogwu, Nneka C Agu, Mercy O Udeh, Joseph A Arusiwon

**Affiliations:** 1 Chemical Pathology, Department of Medical Laboratory Science, Faculty of Health Science and Technology, University of Nigeria, Enugu Campus, Enugu State, Nigeria; 2 Discipline of Clinical Anatomy, School of Laboratory Medicine and Medical Sciences, Nelson R. Mandela School of Medicine Campus, University of Kwazulu-Natal, South Africa; 3 Heamatology, Department of Medical Laboratory Science, Faculty of Health Science and Technology, University of Nigeria, Enugu Campus, Enugu State, Nigeria; 4 Department of Anatomy, Faculty of Basic Medical Sciences, Nnamdi Azikiwe University, Nnewi Campus, Anambra State, Nigeria

**Keywords:** Viral load, HIV/AIDS, human growth hormone

## Abstract

Routine viral load and CD4+ testing is key to monitoring the extent of danger caused by HIV and response to antiretroviral therapy (ART) for HIV individuals, but its availability has been limited in low and middle-income countries. The study sort to ascertain relationship between serum Human Growth Hormone (HGH) gold standard with CD4 cells and viral load in HIV-infected patients. CD4+ T-cells, HIV viral load, and HGH were assayed in HIV- infected patients from May to December 2020. 460 subjects were engaged and separated into two groups: the HIV-infected untreated (Pre-ART) and the control groups. An interventional study was conducted for the Pre-Art group after six months. Serum HGH was assayed by the ELISA method, CD4 cell count was examined by BD-FACScan flow cytometer, and HIV viral load was assessed using RT-PCR. The CD4 count and serum HGH of Pre-ART HIV-infected subjects were significantly low (p<0.05), while the viral load was significantly high compared to those treated with ART for 6months (p<0.05). CD4 count and serum HGH were significantly higher (p<0.05) in females than in males. It also reveals that CD4 count correlates positively with HGH level (r= 0.191**). Serum HGH could serve as a surrogate marker and valuable index in monitoring HIV patients.

## Introduction

Nigeria has the second-highest burden of Human Immunodeficiency Virus (HIV) globally^1^,^2^. In 2019, UNAIDS estimated 3.2 million people were living with HIV in Nigeria, of which 1.1 million (34%) knew their HIV status and 970,000 (30%) were on ART^3^. However, CD4 T cell count and viral load testing are key to monitoring HIV-infected patients^4^,^5^. In April 2020, there were 27 functioning viral load instruments in Nigeria^6^. Each viral load is expected to monitor close to 70,00 patients, with many of those samples transferred from long distances.

Consequently, it resulted in a high transmission and mortality rate due to delayed treatment, poor patient follow-up, and poor adherence to ART. The risk of patients dying from their condition increases in HIV/AIDS-related wasting syndrome, which leads to death when more than 33 percent of the healthy body weight is reduced^7^. Research showed that growth hormone therapy could help overcome the lipodystrophy associated with HIV/ AID^8^. Recombinant Human Growth Hormone was approved In August 1996 by the Food and Drug Administration to treat HIV/AIDS-related wasting syndrome. Moreover, studies have revealed that HGH could be very advantageous in this regard9. Due to its challenges, many middle and low-income countries conduct CD4+ count and viral load tests in a few central laboratories^1^,^6^.

Furthermore, adverse storage and long-distance sample transfer lead to falsely elevated readings, affect the validity of results, probably due to plasma contamination with cell-associated viral nucleic acids, and delay between sample collection, processing, and initial Viral Load (VL) testing^9^. Also, it causes lengthy delays in receiving results and disruptions in the treatment flow^9^. However, the focus of these studies has generally been on identifying cheaper markers that could indicate the presence of high viral load and low CD4+ in people living with HIV on treatment or making decisions about functional impairment and disability. There is an urgent need to adopt a cheaper, dependable, and accurate alternative for viral load/CD4^+^ count, which will be easy to perform in resource-limited settings (RLS). Could the estimation of serum HGH be advantageous since its assay method and equipment required are common and cheaper to operate with shorter processing time when compared with BD FACScan flow cytometer and RT-PCR used by CD4 cell count and HIV viral load, respectively? Furthermore, could serum HGH serve as a substitute for CD4 cell count and viral load assessment during equipment collapses^10^. The study sort to ascertain the relationship between the HGH gold standard with CD4 cells and viral load in HIV-infected patients.

## Materials and Methods

### Area of Study and Study Design

The study was carried out in southeast Nigeria. It is a prospective cross-sectional study involving 460 using a convenience sampling technique: 400 HIV-infected untreated subjects and 60 controlled subjects (non-HIV infected participants), with an interventional study where 391HIV-infected participants were intervened with antiretroviral therapy for six (6) months.

### Subjects Recruitment

All participants were recruited between May 2020 and December 2020 from Chukwuemeka Odumegwu Ojukwu University Teaching Hospital Anambra state, the University of Nigeria, Teaching Hospital Enugu state, Federal Medical Center Ebonyi State, Federal Medical Center Imo State, and Federal Medical Center Abia State. All participants willingly gave their consent before being enrolled in this study.

### Ethical Approval

The ethical approval for this research was obtained from the Ethics committee of Chukwuemeka Odumegwu Ojukwu University Teaching Hospital, Anambra State, and University of Nigeria, Teaching Hospital, Enugu State with Ref: COOUTH/CMAC/ETH.C/VOL.1/ FN:04/0021 and UNTH/CSA/329/VOL5 respectively.

### Inclusion criteria

The study inclusion criteria include male and female participants, already diagnosed HIV infected patients, and control subjects inside the above age bracket (18-60).¬¬¬ Participants with additional health conditions such as liver failure, kidney failure, tuberculosis, heart failure history, and history of harmful alcohol or substance use that could affect the results were not included in the study. Pregnant women and lactating mothers were also excluded.

### Interventional Study

Three hundred and ninety-one (391) HIV infected untreated subjects that participated in the cross-sectional study also gave their consent to continue with the study and thus, were selected for an interventional study for six (6) months on ART (Dolutegravir/Lamivudine/ Tenofovir Disoproxil Fumarate), one tablet per day. Dolutegravir/lamivudine/tenofovir (DTG/3TC/TDF) is a fixed-dose combination antiretroviral medication used to manage HIV/AIDS. It is a combination of dolutegravir, lamivudine, and tenofovir disoproxil. It is the first-line treatment for adults^11^.

### Participant Enrolment

#### Anthropometric Measurements

**Body Mass Index (BMI):** BMI was evaluated using the anthropometric method that Janssen *et al.* 12. Height (m) was measured using a stadiometer, and whole-body weight (kg) was taken using a bodyweight weighing scale with the subject wearing light clothing and without shoes. Body mass index (BMI) was calculated as the ratio of weight (kg) to the square of height (m^2^).

**Blood Pressure Measurement:** Blood pressure (BP)-systolic and diastolic readings were taken from the participants' left arm using a sphygmomanometer (Omron Medical, United Kingdom) after being seated for ten minutes. The reading was taken in the morning to the nearest mmHg.

#### Data Collection Procedure

A standardized questionnaire captures different types of data: epidemiologic information (age, sex, and HIV transmission category), sociodemographic characteristics, and relevant history. Participants who indicated an interest in the study were given a questionnaire to fill out. Those that met the inclusion criteria were thereafter enrolled in the study.

#### Sample Collection Procedure

Venous blood samples of 14.0ml were collected at 15^o^C to 30^o^C from each subject using vacutainer tubes. 9.5ml, 2.5ml, and 2.0ml of the venous blood were dispensed into 10.0ml EDTA (Ethylenediaminetetraacetic acid) sample container, 5.0ml EDTA sample container, and 5.0mls plain sample container respectively and after six months of intervention. The sample containers were labeled with the subject's Laboratory number, age, and sex. The samples in the plain sample container were allowed to stand for 10mins, retracted, and spun for 10 minutes at 3000 rpm, including the whole blood samples in the 10.0ml EDTA containers. The serum and plasma were separated from the red cells using a dry clean Pasteur pipette into a sterile, plain screw-cap specimen container. The viral load assay was not assayed immediately but was stored at -20°C until testing. At the same time, the serum HGH and whole blood CD4^+^ T cell count were estimated immediately after sample collection and processing.

A total of 400 samples were obtained from pre-ART HIV patients at the screening stage, while other samples were collected from the same test subjects the following six (6) months later on ART for the fellow-up test.

#### Analytical methods

The tests were performed in batches daily alongside standards.

#### Analytical methods

The tests were performed in batches daily alongside standards.

#### HGH (Human Growth Hormone)

The serum HGH was estimated using Enzyme-Linked Immunosorbent Assay with a kit assay system^13^.

#### Principle

The quantitative HGH test is based on the principle of a solid phase enzyme-linked immune-sorbent assay. The assay system utilizes a polyclonal anti-HGH antibody for solid phase (microtiter wells) immobilization and another mouse monoclonal anti-HGH antibody in the antibody-enzyme (horseradish peroxidize) conjugate solution. The test sample (serum) is added to the HGH antibody-coated microtiter wells together with the enzyme conjugate reagent. HGH molecules are sandwiched between the solid phase and enzyme-linked antibodies. After incubation at room temperature, the wells are washed with HGH wash solution to remove unbound labeled antibodies. A solution of TMB is added and incubated for 20minutes resulting in the development of a blue colour. The colour development is stopped with the addition of 2N HCL, and the colour changes yellow. Finally, the absorbance is measured spectrophotometrically at 450nm. The concentration of HGH is directly proportional to the colour intensity of the test sample.

#### Analytical procedure

All reagents were brought (Microplate wells) to room temperature (18 – 22^o^C) before use. The Concentrated Wash solution was diluted 1volume of wash buffer concentration with 49volumes of distilled water. Pipetted 50µLstandard (S_0_ – S_1_), Serum samples, and control were put into the desired number of coated wells and fixed in the holder. Enzymes Conjugate (100 µL) were added into the same coated wells. It was mixed efficiently and incubated at room temperature for 60minutes; the incubated mixture was removed and was washed 5times with diluted wash buffer. The wells were struck sharply onto absorbent material (paper towel) to remove residual water droplets.

TMB – substrate (100µL) was pipetted into all the wells, gently mixed for 5seconds, and incubated at room temperature for 20minutes; the incubated mixture was removed and washed 5times with diluted wash buffer. Stop solution (100 µL) was added into the same coated wells, gently mixed for 30seconds, and read the absorbance at 450nm with a microplate within 30mintues.

#### Reference Range

0 – 7.0ng/mL^13^.

#### CD4^+^ Count Assay

The whole blood CD4 was estimated using BD FACScan flow cytometer with kit assay system (BD Biosciences, 2005). The reference range used was 500 cell/uL and above^14^.

#### Principle

When whole blood is added to the reagent, fluorochrome-labeled antibodies bind specifically to lymphocyte surface antigens; after a fixative solution is added to the reagent tubes, the sample is run on the instrument. Here, the cells contact laser light, which causes the fluorochrome-labeled cells to fluoresce^14^.

**Analytical procedure (Running patient samples):** The reagent pairs were vortexed upright for 5 seconds. The CD4 tubes were uncapped, and the reagent pairs were placed in the sample holder. The RUN key was pressed, the reagent pairs were removed, and the CD4 tubes were recapped. The CD4 tubes were uncapped, and the reagent pair was placed in the sample holder, so the CD4 tube was in the run position, and the RUN key was pressed^14^.

#### HIV Viral Load/ HIV RNA quantification (Abbott RealTime HIV-I Kit)

The HIV viral load was estimated using the Reverse Transcription Polymerase Chain Reaction (RT-PCR) method based on quantitation of Human Immunodeficiency Virus type 1 in human plasma from HIV-infected individuals with kit assay system^15^. The reference range used was 0 - 1000 copies/mL^15^,^16^,^17^

**Principle:** The Abbott Realtime HIV Assay uses RT-PCR to generate amplified products from the RNA genome of HIV in clinical specimens. An RNA sequence unrelated to the HIV target sequence is introduced into each specimen at the beginning of sample preparation. This unrelated RNA sequence is simultaneously amplified by RT-PCR and serves as an internal control (IC) to demonstrate that the process has proceeded correctly for each sample. The amount of HIV target sequence present at each amplification cycle is measured using fluorescent-labeled oligonucleotide probes on the Abbott m2000rt Analyzer. The probes do not generate signals unless they are specifically bound to the amplified product. The amplification cycle at which the Abbott m2000rt detects fluorescent signal is proportional to the log of the HIV RNA concentration present in the original sample.

**Analytical procedure:** Cells were washed and re-suspended in DNA-STAT 60 (Tel-Test) to extract genomic DNA (the assembly of the amplification master mix and sample elutes into the Abbott 96well optical reaction plate). A dedicated pipette dispenses 50uL aliquots of the amplification master mixed into the Abbott 96-Well Optical Reaction Plate in the amplification area. The Abbott 96-Well Optical Reaction Plate was transferred to the Eppendorf PCR cooler in the sample preparation area, centrifuged for 5minutes, and transferred to the Amplification Area, where Abbott 96-Well Optical Reaction takes place in the Abbott m2000rt Analyzer. The sample volume being tested was selected from the Protocol screen, then Abbott Real-time HIV protocol was initiated. The concentration of viral HIV RNA in a sample and control is calculated from the Abbott m2000rt Analyzer^15^.

### Statistical Analysis

The statistical analysis was performed using the paired student's t-test, and correlation studies were performed using Pearson's correlation coefficient. Statistical package for social sciences (SPSS) 21.0 was used for the statistical analysis. Differences between sex groups were assessed using Analysis of Variance (ANOVA) with Post Hoc (Non-parametric) comparison for intergroup variability. The comparison was made at a 95% confidence level. A p-value equal to or less than 0.05 (p ≤ 0.05) was used as a threshold for statistical significance.

## Results

All results are Mean ± Standard Deviation. The abbreviation NS was used to denote no statistical significance, while S was used to denote statistical significance difference.

The CD4 counts, viral loads, and serum HGH levels presented in table 1 showed significant differences (p< 0.05) between Pre-ART (Baseline) patients and HIV-infected patients treated with ART for 6 months. There were also significant differences (p< 0.05) in the CD4 count and serum HGH level of controls when compared with the CD4 counts and serum HGH levels of Pre-ART (Baseline patients and HIV-infected patients treated with ART for 6 months.

**Table 1:** CD4 count, viral load, and serum HGH level in baseline (Pre-ART) HIV infected patients, HIV infected patients on six months ART and control in the study area (mean ± SD)

Group	CD4 Count (cell/uL)	Viral load (copies/mL)	HGH level (ng/mL)
**Baseline (A)**	353.22 ± 170.42	184313.68 ± 534610.94	0.40 ± 0.36
**ART (B)**	422.97 ± 258.86	2731.70 ± 8693.07	0.82 ± 4.47
**Control (C)**	1078.67 ± 29 7.12	–	1.99 ± 1.09
**P – value ^(AvsC)^**	**0.01 (S)**	–	**0.04 (S)**
**P – value ^(AvsB)^**	**0.01 (S)**	**0.01 (S)**	**0.01 (S)**

Concerning sex, viral loads of Pre-ART (Baseline) patients and HIV-infected patients showed no significant difference (p= 0.96; p= 0.39) between males and females. However, CD4 counts and serum HGH levels of all study groups were observed to be significantly higher (p <0.05) in females (Table 2)

**Table 2:** Sex distribution of CD4 count, viral load, and serum HGH level in baseline (Pre-ART) HIV-infected patients, HIV-infected patients on six months ART, and control subjects in the study area (mean ± SD)

Groups	Parameters	Female	Male	t-value	P-value
**Baseline**	**CD4 Count**	389.65 ± 180.94	316.06 ± 150.59	3.90	0.01 (S)
**(Female: 202; Male: 198)**	**Viral Load**	184988.93 ± 608212.29	182714.63 ± 447380.68	0.04	0.96 (NS)
	**HGH level**	1.25 ± 6.26	0.37 ± 0.15	1.98	0.04 (S)

**ART**	**CD4 Count**	474.98 ± 315.77	3369.91 ± 168.46	4.14	0.01 (S)
**(Female: 199; Male:** 192)	**Viral Load**	1854.14 ± 5536.44	3622.57 ± 10953.12	-2.04	0.39 (NS)
	**HGH level**	0.53 ± 0.46	0.27 ± 0.13	7.94	0.01 (S)

**Control**	**CD4 Count**	1169.65 ± 302.85	896.70 ± 183.30	3.70	0.01 (S)
**(Female: 40; Male: 20)**	**HGH level**	2.32 ± 1.26	1.35 ± 0.68	3.53	0.01 (S)

Correlations between all parameters for the three subject groups in this study were presented in table 3. The correlations between serum HGH levels and CD4 counts were significantly positive for all study groups. However, a significant negative correlation was observed between serum HGH levels, viral loads, and CD4 counts when compared in the Pre-ART (Baseline) patients and HIV-infected patients on six months ART.

**Table 3:** Correlations of CD4 count, viral load, serum HGH level in baseline (Pre-ART) HIV infected subjects, HIV infected subjects on six (6) months ART, and control subjects in the study area

Parameters	Group	n	r - value	P - value
HGH vs CD4 Count	Baseline	400	0.191*^(r)^	0.001*
Viral Load vs CD4 Count	Baseline	400	-0.324**^(r)^	0.001*
HGH level vs Viral Load	Baseline	400	-0.116**^(r)^	0.001*
HGH vs CD4 Count	ART	391	1.30**^(r)^	0.002*
Viral Load vs CD4 Count	ART	391	-0.228**^(r)^	0.001*
HGH level vs Viral load	ART	391	-0.106*^(r)^	0.001*
HGH level vs CD4 Count	Control	60	0.111^**(r)^	0.001*

## Discussion

The prevalence of HIV in Nigeria remains the second to the highest epidemic and has the fourth to largest HIV & TB co-infection globally. The low levels of treatment and care for HIV-infected individuals remain persistent, leading to many AIDS-related deaths. Many middle and low-income countries conduct CD4^+^ count and viral load tests, which are supposed to be the cornerstone and key to monitoring individuals living with HIV in a few central laboratories due to their complexity and cost^4^. This work sets out to pinpoint a cheaper laboratory test that could reveal the degree of immune depletion and restoration of immunodeficiency. According to data generated from this study, there was a statistically significant decrease in CD4 cell count and HGH in baseline (Pre-ART) subjects with HIV (p< 0.05) when compared with the control subjects. This could result from rapid loss of CD4 cells, immune depletion or immunodeficiency, and susceptibility to opportunistic infection^18^. The observed low level of serum HGH was in line with those reported by James and Dennis^19^, which may be due to weight loss, diarrhea or chronic weakness, immune dysfunction, atrophied thymus gland, fever, and fat redistribution associated with HIV. Consequently, in HIV infection, the body consumes more energy reserves^20^.

The CD4 cell count and HGH in HIV baseline (Pre-ART) subjects were significantly low (p< 0.05). At the same time, the viral load level showed a higher significant difference (p< 0.05) in baseline (Pre-ART) subjects when compared with HIV patients on 6months ART, as shown in Table 1. The observed high CD4 count in HIV patients on 6months ART supported the findings of those reported by WHO^6^. They explained in general that antiretroviral therapy (ART) had improved the health of more than 18 million people infected with HIV by controlling viral replication, reducing the risk of the degree of immunosuppression and transmission due to the damage done to the immune system, especially by destroying the white blood cells known as CD4 lymphocyte cells^3^.

The high level of serum HGH in HIV patients on 6months ART when compared with when the baseline (Pre-ART) patient could be due to loss of muscle mass, fat redistribution, loss of lean body mass, rebounding virus, metabolic and morphologic changes, known as HIV associated lipodystrophy syndrome (HALS) that affects approximately 40 % of HIV infected patients^9^,^20^. The observed reduced serum viral load in HIV patients on 6months ART could be because ART blocks replication in short-lived CD4 cells that are actively infected^21^.

In this study, the female in all groups showed high statistically significant differences (p< 0.05) in CD4 cell count and serum HGH compared with the male patients' counterparts. Nevertheless, viral load levels showed no statistically significant changes (p> 0.05) in their sex distribution, as shown in Table 2. The observed high CD4 count in female baseline (Pre-ART) HIV subjects when compared with that of male counterparts were in agreement with those reported by Jensen-Fangel *et al.*^12^ and Afolabi *et al.*^22^, who explained in general that females have higher CD4 cell counts than male, whether HIV-infected or not. The high CD4 cell observed in the present study suggests that females repopulate their peripheral CD4 cells in response to virus suppression more quickly than males. The high serum growth hormone (GH) concentrations observed in women supports the work of Pincus *et al.*^23^. They reported that females secrete growth hormone with more pprocess irregularity than males, implying that modes of GH secretion are regulated differently in males and females. But the neuroendocrine mechanisms that underlie such sex differences are unknown^23^.

Moreover, the serum HGH showed a significant positive correlation with CD4 count and a significant negative correlation with the viral load, as shown in Table 3. This shows that low-level serum HGH is associated with a significant loss in lean body mass, wasting- mostly common in the advanced stages of AIDS, HIV associated adipose redistribution syndrome (HARS), decline in the chances to fight infections, reducing the rate of survival in baseline (Pre-ART) patients and subjects on antiretrovira^19^,^20^,^24^,^25^,^26^. In addition, Lori *et al.*^27^ reported that GH treatment enhanced thymic output, as measured by the frequency of T cell receptor rearrangement and the numbers of circulating total CD4+ T cells. These findings provide compelling evidence that GH induces increased T-cell production and may facilitate CD4+ T cell recovery in HIV-1–infected adults^27^,^28^. Generally, estimation of HGH could serve as a diagnostic and prognostic tool in HIV/AIDS management since their method has a significantly reduced processing time, low cost, simpler, faster, and also serve as an alternative or surrogate marker to CD4 cell count and viral load estimation in case of machine breakdown or serve as a solution to the topical problems hindering the conduct of the CD4 cell count and viral load testing.

Disease^29^ that reported that the prevalence and incidence data of low GH vary widely due to the lack of standard diagnostic criteria^30^. However, the National Cooperative Growth Study (NCGS), Genentech's now closed large North American database, revealed that 85% of patients receiving GH treatment for idiopathic GHD were white, 6% were black American, and 2% were Asian^29^.

## Conclusion

The research findings suggest that HGH can serve as a substitute or surrogate marker to CD4 cell count and HIV viral load, especially in hard-to-reach areas where samples transfer to viral load testing laboratories is very challenging or during equipment collapse. This is because our study showed a strong correlation between CD4+ T cell count and serum HGH level, as well as a strong correlation between HIV viral load and serum HGH, which also reflects the production rate of HIV virions and the rate of CD4 cell destruction.

## Recommendation

The Nigerian/State National algorithm for managing and treating HIV/AIDS should include serum HGH determination, particularly in low- and middle-income locations. The Nigerian/State National algorithm for managing and treating HIV/AIDS should include serum HGH determination, particularly in low- and middle-income locations.

## Abbreviations

HGH: Human Growth Hormone
AIDS: Acquired Immune Deficiency Syndrome
ART: Anti-Retroviral Treatment
BD FAScan: Becton Dickinson FASCan
HIV: Human Immunodeficiency Virus
RT-PCR: Reverse Transcript- Polymerase Chain Reaction
